# Acute response in circulating microRNAs following a single bout of short-sprint and heavy strength training in well-trained cyclists

**DOI:** 10.3389/fphys.2024.1365357

**Published:** 2024-03-12

**Authors:** Anita Ryningen, Kari Rostad, Elisabeth Ersvær, Gry Sjøholt, Gøran Paulsen, Hilde Gundersen, Morten Kristoffersen, Lise Bjørkhaug

**Affiliations:** ^1^ Department of Safety, Chemistry and Biomedical Laboratory Sciences, Western Norway University of Applied Sciences, Bergen, Norway; ^2^ Department of Biotechnology, Inland Norway University of Applied Sciences, Lillehammer, Norway; ^3^ Department of Sport, Food and Natural Sciences, Western Norway University of Applied Sciences, Sogndal, Norway; ^4^ Department of Physical Performance, Norwegian School of Sport Sciences, Oslo, Norway; ^5^ Department of Sport and Physical Activity, Western Norway University of Applied Sciences, Bergen, Norway

**Keywords:** cycling, heavy strength training, short-sprint training, MicroRNAs, recovery

## Abstract

**Background:** Heavy strength (HS) and short-sprint (SS) are commonly used training methods for competitive road cyclists, with the aim to improve the anaerobic power and short time cycling performance. Knowledge of how such training methods affects biochemical as well as molecular factors, are particularly important for determining individual recovery and long-term adaptations. The primary aim of the current study was to investigate the expression levels of small non-coding RNAs in response to HS and SS training in elite cyclists as potential biomarkers for individual optimal restitution time.

**Methods:** Eleven well trained cyclists performed one session of HS training and one session of SS training on separate days. Blood samples were taken at baseline and 5 min, 1 h and 21 h post training. Along with physiological measurements and biochemical factors (serum creatine kinase, myoglobin, human growth hormone and plasma lactate), real-time quantitative PCR was used to explore whether HS and/or SS training influenced the abundance of 24 circulating miRNAs, in serum, associated with muscle development, angiogenesis, and/or inflammation.

**Results:** Based on complete miRNA profiles from nine cyclists, the miRNAs showing most altered expression after both training sessions included the three striated muscle-specific miRNAs (myomiRs) miR-1-3p, 133a-3p and 133b-3p. While all three miRNAs showed significantly highest expression at 1 h post HS session, the acute effect of the SS session included a significantly higher level of miR-1-3p alone, at 5 min (highest), as well as at 1 h and 21 h post session. Correlation (negative) with biochemical markers was only shown for miR-133a-3p and CK (r = −0.786, *p* = 0.041) and between miR-133b-3p and [La^−^] (r = −0.711, *p* = .032), at 21 h post SS session.

**Conclusion:** Our findings support that unique myomiRs are regulated by HS and SS training. Such knowledge may be important for individually adjusted restitution times.

## Introduction

Competitive road cycling is an endurance sport which demands sustained aerobic power outputs (Jeukendrup et al., 2000). However, high-intensity efforts and sprinting abilities are important to manage sudden breakaways, to close gaps to riders ahead, and in a fast finishing sprint (Menaspa et al., 2015). Thus, many cyclists also include heavy strength (HS) training and specific short sprint (SS) training, with the aim of improving sprinting power.

Both HS and SS training challenges the neuromuscular system to generate maximal power, by different combinations of load and velocity. While HS is performed with high loads and slow concentric and eccentric muscle actions, cycling-specific SS training applies lower load and higher velocity muscle contractions (Kraemer et al., 2004; Martin, 2007). Although the effects of HS training on cycling performance have long been debated, an increase in maximal force (Bertucci et al., 2005; Skovereng et al., 2018; Del Vecchio et al., 2019) and muscle hypertrophy (Ronnestad et al., 2010; Vikmoen et al., 2016) has been shown in trained cyclists, after adding HS training to their usual endurance training, with positive effects on anaerobic power and short time cycling performance. Unlike HS training, the effect of SS training on cycling performance has been less studied, possibly due to the lack of well-established training methods and debate on how to measure sprint performance. Knowledge regarding the acute effect of systematic HS and SS training on sprint performance, aerobic capacity and recovery time, is undoubtedly valuable for both athletes, coaches and researchers. It is also important to gain knowledge about physiological, biochemical and metabolic factors involved in individual recovery and long-term adaptions after such training.

Exercise *per se* induce changes in gene expression, which affect skeletal and myocardial metabolism and regeneration, as well as mitochondrial synthesis, angiogenesis and inflammation (Gundersen, 2011; Egan and Zierath, 2013; Camera et al., 2016; Hughes et al., 2018). One of the molecular co-players in gene expression and modulation of signal propagation are the microRNAs (miRNA). MiRNAs are small (19–22 nucleotides), endogenous, non-coding RNA molecules that regulate gene expression by repressing specific target genes either by translational inhibition or mRNA degradation (Krol et al., 2010). The potential role of miRNAs in communication between cells and tissues is strongly supported by the fact that miRNAs can be exported and imported by cells through mechanisms involving vesicle trafficking and protein carriers. Circulating blood plasma miRNAs (c-miRNA) are associated with microparticles (Rayner and Hennessy, 2013), exosomes (Valadi et al., 2007), apoptotic bodies (Zernecke et al., 2009), proteins (e.g., Argonaute) (Li et al., 2012), or high-density lipoproteins (Vickers et al., 2011). Further, miRNAs are actively or passively secreted into human biofluids where they can have systemic effects (Kosaka et al., 2013; Shah and Calin, 2013). As miRNAs are quite stable molecules (Tsui et al., 2002; Mitchell et al., 2008) and easily accessible via blood samples, c-miRNAs represent interesting biological markers associated with the physiological responses to exercise and adaption (Horak et al., 2018; Sapp et al., 2019). Circulating miRNA are found to be regulating proliferation, differentiation, hypertrophy, and nutrient metabolism in skeletal muscle functions (Domanska-Senderowska et al., 2019), in angiogenesis, inflammation (Li et al., 2018), and in metabolic change (Massart et al., 2021). Muscle induced c-miRNAs and c-miRNA biomarkers for endothelial damage have been shown altered in response to acute aerobic exercise (Nielsen et al., 2014; Uhlemann et al., 2014). Also, c-miRNAs playing a role in strength training-induced muscle adaptation and as biomarkers for muscle damage have been shown altered in response to resistance exercise session (Sawada et al., 2013; Uhlemann et al., 2014).

In a recent study, we found that the acute responses following HS and SS training sessions in trained cyclists differ, whereby such training sessions differentially affect physiological and biochemical markers of metabolic stress and muscle damage, in serum, relevant for individual recovery and long-term adaptations (Kristoffersen et al., 2019). In this study, we thus followed up by investigating miRNAs associated with metabolic homeostasis. More specifically, 24 c-miRNAs with expected functional regulation in skeletal and heart muscle, hypoxia and angiogenesis, and in metabolism and inflammation, listed in Supplementary Table S1, were profiled from the same homogeneous group of young male elite cyclists (Kristoffersen et al., 2018), acutely after performing a SS and an HS training session. We aimed to explore (1) differences in c-miRNA profile responses to SS and HS training sessions, and (2) correlation between unique c-miRNAs and biochemical markers of muscle metabolism and damage (lactate, human growth hormone, creatine kinase and myoglobin). Our major/overall goal was to evaluate specific c-miRNAs as potential markers of recovery for long-term adaptions to training in well-trained athletes, based on their acute response post HS and SS training.

## Materials and methods

### Participants

Eleven well-trained cyclists (males), adept with HS and SS training from daily training routines, gave their written informed consent to participate in the study. Criteria fulfilled for participation included: i) doing competitive cycling at national or international level, ii) having maximal oxygen uptake (*VO*_2max_) of ≥60 mLּ kg^-1^ ּּ min^-1^, iii) having implemented strength training, including squat, hip flexion and leg press, twice a week for a minimum of 4 weeks before testing, and iv) being currently healthy and free from injury. Baseline physiological characteristics of nine of the eleven study participants where reliable and shown in Table 1, and complete miRNA profiles were provided. The study was evaluated by The Regional Committee for Medical and Health Research Ethics in West Norway to not include any medical or health related ethical concerns. The study was then approved by The Norwegian Data Protection Authority (45048).

**TABLE 1:** Physiological characteristics of the male cyclists included in the study (*n* = 9).

Age (years)	17.8 ± 1.7
Body height (cm)	182.2 ± 6.6
Body mass (kg)	72.0 ± 4.7
Body fat (%)	11.4 ± 3.2
VO_2_max (ml ּ kg^−1^ min^−1^)	66.4 ± 2.5
Power output at [La−] of 4 mmol·L^−1^ (W)	285 ± 25

### Study design

**FIGURE 1:**
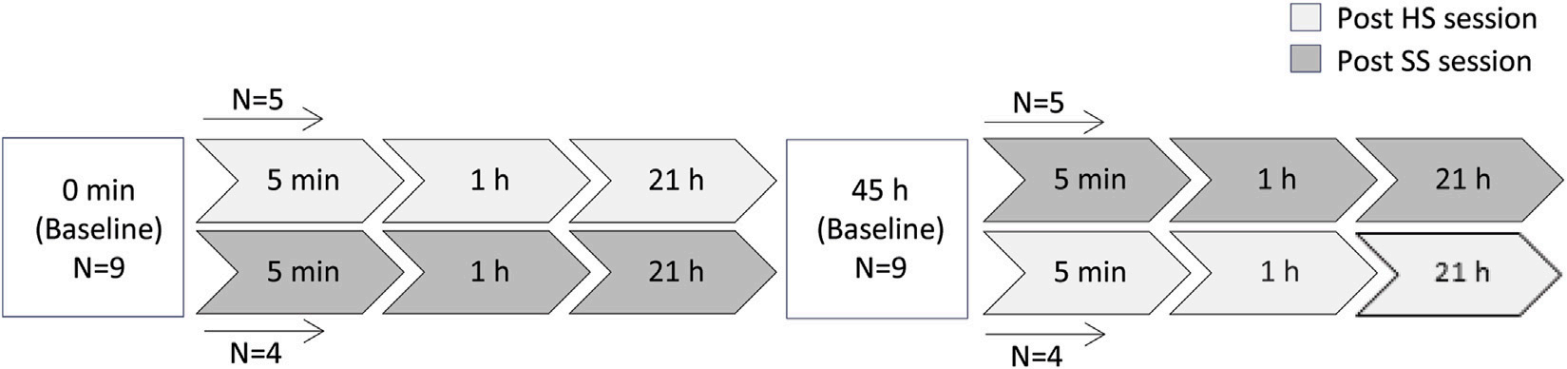
An overview of the study design/experimental setup and timepoints pre/post HS and SS training sessions. N indicates the number of individuals performing in each training session. HS and SS indicate heavy strength training and specific short sprint training, respectively.

The study was performed with a crossover design. Participants were randomly divided into two groups, and the HS and SS training sessions were conducted in a randomized order. 48 h passed between the two sessions for both groups. Blood samples were collected before breakfast (baseline/0 h) and at 5 min, 1 h and 21 h post training sessions (Figure 1).

### Preliminary testing and familiarization session

Approximately 14 days before the first experimental training session, a submaximal and maximal test on a cycle ergometer (Lode Excalibur Sport, Groningen, Netherlands) was completed by the participants, as described by Kristoffersen et al. (2018). Familiarization to the specific sessions used in the present study was performed 1 week before the first experimental session. The 6RM load for each exercise in the HS session was defined during the familiarization session for each participant. During the cycling familiarization session, the pedaling resistance applied for the sprints was individually adjusted using an air braked bicycle ergometer (WattBike, WattBike Ltd, Nottingham, UK). This bike was used for both familiarization and experimental trials. To ensure that the participant achieved the highest possible power output during the 8-s sprints at a cadence of 130–140 revolutions per min (RPM) (Hopker et al., 2010), each participant performed at least three sprint at different resistance level with 2 min recovery in between.

### Experimental procedures

In order to perform the same volume of low-intensity training during the data collection period, all participants were instructed to abstain from strenuous exercise. They were also instructed to have similar diet 48 h prior to both experimental training sessions. All participants registered daily meals, physical activity and sleep, during the data collection period. Prior to the HS and SS training sessions, individuals arrived to the laboratory for the baseline blood sample at the same daily time after an overnight fast of at least 8 h. Participants were served a standardized breakfast 1.5 h prior to each training session.

### HS and SS training sessions

The HS and SS sessions were carefully designed to mirror typical training sessions used by Norwegian world-class cyclists. The total duration of the SS and HS session were approximately 45 min including warm-up.

The SS session consisted of three sets of four 8 s intervals with maximal effort, from a standstill start in a seated position (individually chosen preferred leg). Each interval repetition was spaced by a 2 min active recovery, and each sprint set by a 5 min active recovery. Active recovery included cycling at 70% of HR_max_. A cycle ergometer (WattBike, WattBike Ltd, Nottingham, UK) was used for all SS sessions, and allowed measurement of power output (Hopker et al., 2010).

The HS sessions included 3 sets of 6 repetition maximum (RM) per exercise. Sessions consisted of two-legged squats in a smith machine (TKO, Houston, USA), unilateral leg-press (Mobility, Norway), and unilateral hip flexor exercises in a cable cross apparatus (Gym 80, Gleskirchen, Deutschland). Three min recovery was given between sets and 5 min between each exercise. Participants were instructed to carry out the concentric phase with maximal effort. The eccentric phase was instructed completed as a controlled movement lasting for 2 s.

### Blood sampling and serum analyses

Blood samples were collected pre-exercise (baseline, in fasted state) and at 5 min, 1 h, and 21 h post the HS and SS training session. Blood was sampled from an antecubital vein into vacutainers containing clot activator for serum separation (BD Vacutainer SST II Advance Plus Blood Collection Tubes) and were kept at room temperature for 30 min prior to centrifugation (1,300 x g for 10 min). Serum for miRNA analyses was aliquoted to Nunc cryotubes and subsequently stored at −80°C until analyzed.

### Biochemical marker levels

Serum creatine kinase (CK), myoglobin (Mb), human growth hormone (hGH) and plasma lactate ([La^¯^]) was analyzed as previously described (Kristoffersen et al., 2018). Biochemical marker levels at specific time points post training session are presented in Supplementary Figure S2.

### TaqMan low density array (TLDA) assay

For the TLDA assay, total RNA was extracted from 100 μL serum samples using the MagMAX *mir*VANA Total RNA Isolation Kit (Applied Biosystems, Foster City, CA) and according to the manufacturers’ instructions. Eluted RNA (50 μL) was stored at −20°C until further analysis. For oligo RNA spike-in controls (oligo-cel-miR-54-3p, -238-3p and -39-3p), 5 μL 0.2 nM spike-in RNA oligos was added to 100 μL serum samples to a final concentration of 10 pM prior to RNA extraction. cDNA synthesis was performed using the TaqMan Advanced miR cDNA synthesis kit (Applied Biosystems, Foster City, CA) and according to the manufacturers’ instructions. Mature miRNAs were firstly modified in a poly(A) tailing reaction using 2 μL total RNA in the reaction mix (0.5 μL 10X Poly(A) buffer, 0.5 μL ATP, 0.3 μL Poly(A) enzyme, and 1.7 μL nuclease free water), followed by adding 5 μL into the subsequent adaptor ligation reaction mix (3 μL 5X DNA Ligase buffer, 4.5 μL 50% PEG 8000, 0.6 μL 25X Ligation Adaptor, 1.5 μL RNA Ligase, and 0.4 μL nuclease free water). For the reverse transcription reaction, equal volumes (15 μL) of reaction mix (6 μL 5X RT Buffer, 1.2 μL 25 mM dNTPs, 1.5 μL 20X Universal RT Primer, 3.0 μL 10XRT Enzyme Mix, and 3.3 μL nuclease free water) and adapter ligation reaction product was used.

miRNA profiling was performed using the TLDA custom-configured TaqMan Array Cards (Applied Biosystems, Waltham, USA). In order to increase the sensitivity of the TLDA, a preamplification was performed after the reverse transcription using a miR-Amp reaction mix (25 μL 2X miR-Amp Master Mix, 2.5 μL 20X miR-Amp Primer Mix, and 17.5 μL nuclease free water) with 5 μL modified mature miRNAs. For the real-time PCR reactions, 25 μL of a 1:10 dilution of the cDNA template (miR-Amp reaction product) was used in the PCR reaction mix also including 50 μL 2X TaqMan Fast Advanced Mastermix and 25 μL RNase free water. 100 μL PCR reaction mix was applied to each port of the TLDA-card resulting in a 1 μL volume in each well.

The 7900HT Fast Real-Time PCR System (Applied Biosystems, Foster City, CA) was used for the real-time PCR run and all reactions were performed as specified in the protocol of the manufacturer (TaqMan Advance miRNA assays User Guide. TaqMan Array Cards (MAN0016122)). Properties of the run was Comparative Ct (ΔΔCt) and fast cycling mode. miRNA concentrations were analyzed using the ExpressionSuite software (Applied Biosystems, Foster City, CA) and for the normalization of miRNA expression levels, the average C_*t*_ values of all miRNAs (global normalization) was used. The fold changes of miRNA expression were calculated using the eq. 2-ΔΔCq.

### Statistical analyses and software

To obtain comprehensive miRNA expression profiles at various time points following the HS and SS training sessions, we examined the expression profiles of 24 miRNAs in blood serum from the nine elite cyclists. To identify differences in the expressions of miRNAs in the training session groups, compared to baseline expression, we performed a volcano plot filtering against these miRNA expression profiles at specific post session time points. Clustering analysis of the expression patterns of microRNAs was performed using ExpressionSuite software.

One-way ANOVA analyses were performed on the LOG (2^ -(ΔΔ-Ct)) values to evaluate differences between baseline and 5 min, 1 h and 21 h following both the HS and the SS training session for each of the three miRNAs miR-1-3p, miR-133a-3p and miR-133b-3p. (A Shapiro-Wilk analysis was firstly performed and confirmed that the data were normally distributed). Least Significant Difference (LSD) *post hoc* tests used for further comparisons.

Relationships between levels of miRNAs and biochemical markers ([La], CK, Mg, hGH) after HS and SS training sessions were assessed using Pearson’s correlation coefficient analysis (Batterham and Hopkins, 2006). An r-value between .01 and .29 was defined as a small correlation, between 0.30 and 0.49 as a medium correlation, and from 0.5 to 1.0 as a large correlation (Cohen, 1988). The Statistical Products of Service Solution package (SPSS Statistics, version 29) was used for all statistical analyses. For all analyses, statistical significance was determined as <0.05. For the correlation analyses, confidence intervals (CI) are presented.

## Results

### Differentially expressed miRNAs after HS and SS training sessions

**FIGURE 2:**
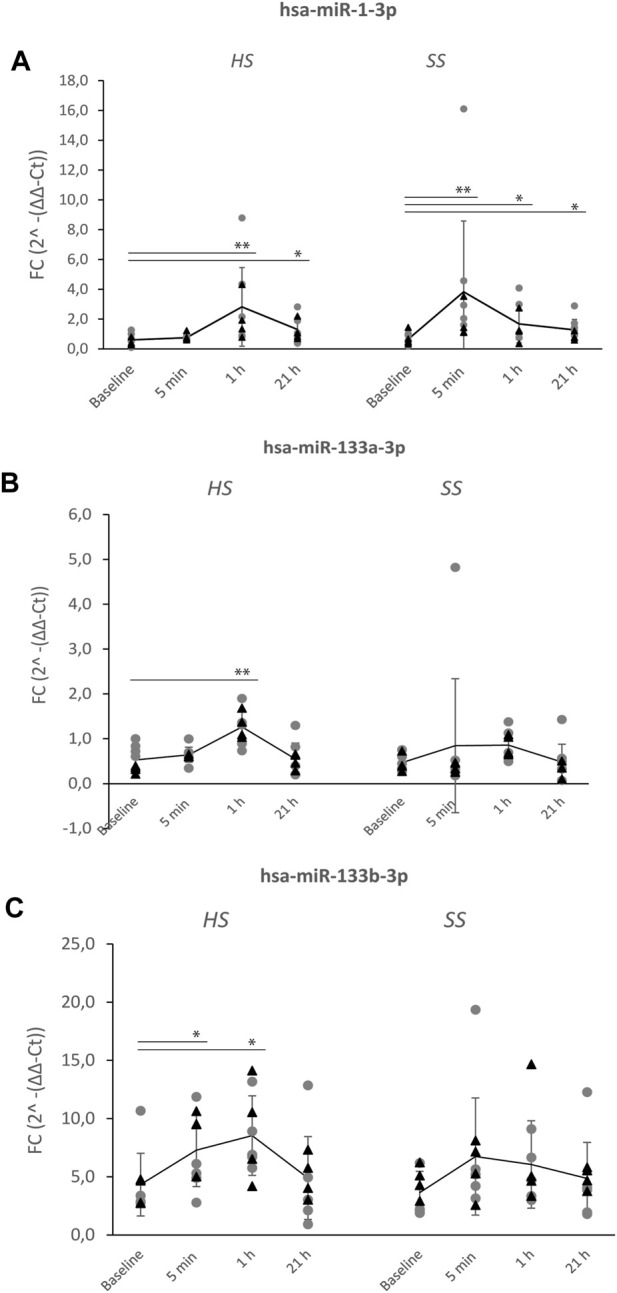
Levels of upregulated hsa-miR-1-3p, hsa-miR-133a-3p and hsa-miR-133b-3p after the HS and SS training sessions. Serum concentrations of three miRNAs were measured following a heavy strength training session (HS) and a short-sprint training session (SS) pre-(baseline) and post-exercise (5 min, 1 h and 21 h) in nine well-trained cyclists (Figure 1). Significant differences after HS and SS sessions of **(A)** hsa-miR-1-3p, **(B)** hsa-miR-133a-3p, **(C)** hsa-miR-133b-3p, compared to baseline levels, are indicated as *p < 0.05, **p < 0.01. Mean levels (line-graph) and standard deviations (vertical lines) are shown for each time point. Gray dots (●) shows miRNA levels of individuals starting with HS > SS session, while black triangles (▲) shows miRNA levels of individuals starting with SS > HS session.

The overall expression profiles of the 24 serum miRNAs in the nine elite cyclists was firstly performed by unsupervised hierarchical clustering of all miRNAs, for all timepoints after the SS and HS training sessions (Supplementary Figure S1). We followed up by investigating levels of three unique miRNAs expressions at individual time points after HS and SS training sessions (Figure 2).

One-way ANOVA analyses showed significant main effects in Fold Change 2^-(ΔΔ-C*t*) values for the three miRNAs comparing before and after the HS training session (has-miR-1-3p: F(3,32) = 7.469, *p* < 0.01, has-miR-133a-3p: F(3,32) = 8.828, *p* < 0.01 and has-miR-133b-3p: F(3,31) = 4.068, *p* < 0.015). A *post hoc* (LSD) test showed significantly higher levels of all three miRNAs at 1 h post HS session (has-miR-1-3p and hsa-miR-133a-3p by *p* < 0.01 and has-miR-133b-3p by *p* = 0.011). Has-miR-1-3p levels alone was also significantly increased at 21 h post HS session (*p* = 0.013) (Figures 2A–C).

A significant main effect before and after the SS training session was only found for has-miR-1-3p (F(3,32) = 7.115, *p* < 0.001). The *post hoc* test showed significantly higher levels of this miRNA already at 5 min (*p* < 0.001), at 1 h (*p* < 0.015) and at 21 h (*p* = 0.035).

**FIGURE 3:**
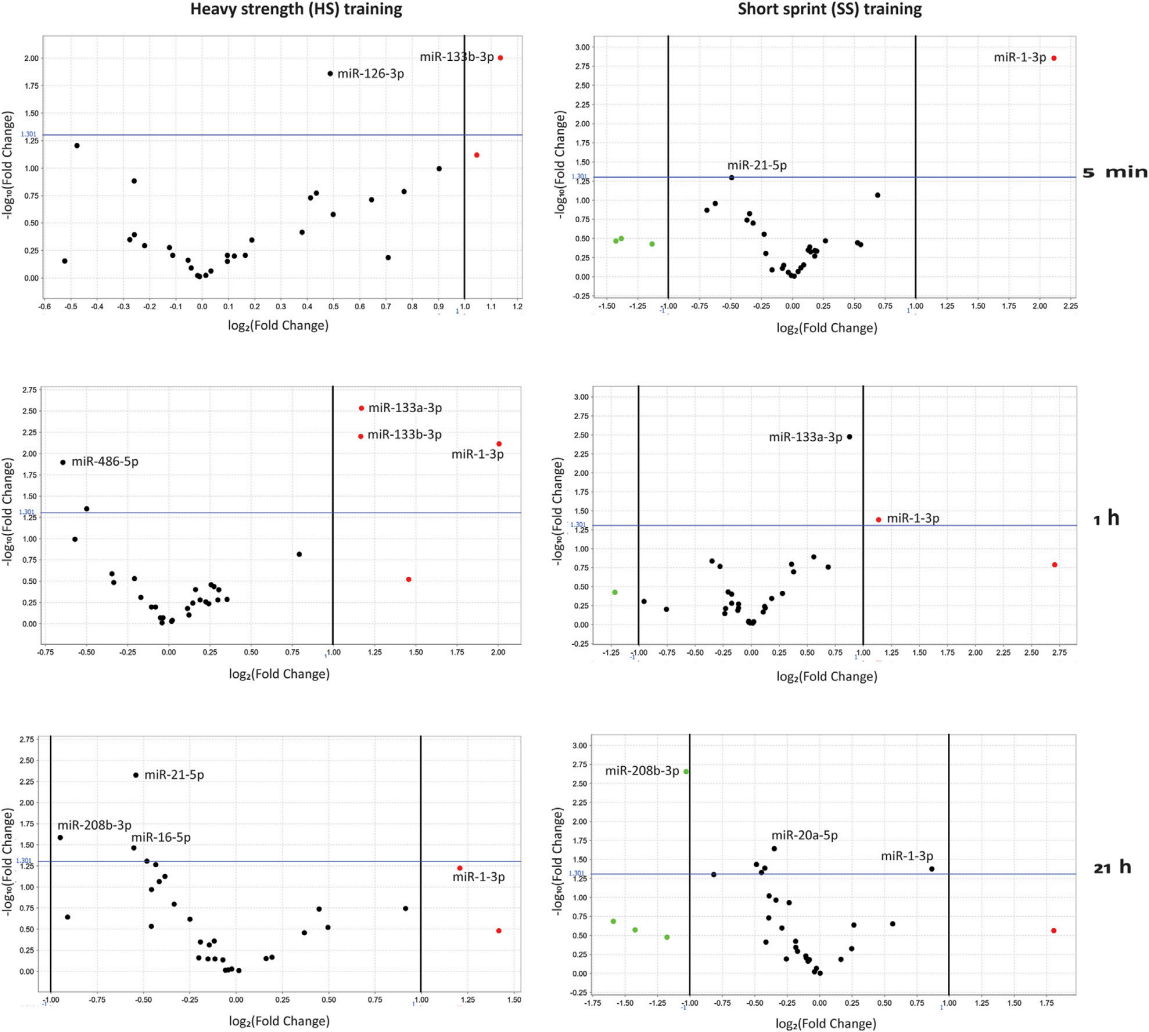
Volcano plots of differentially regulated miRNAs at 5 min, 1 h and 21 h post HS (heavy strength) and SS (short sprint) training sessions, normalized to miRNA baseline levels. A change in miRNA expression was considered statistically significant if the Fold Change (FC) was >2.0 and the *p*-value was <0.05.

Analysis of altered expression patterns of the 24 miRNAs (including the 4 control miRNAs and 3 spike-in miRNAs) at various time points post HS and SS training session is also presented by Volcano plots (Figure 3), by their log_2_(Fold Change), compared (normalized) to session specific baseline miRNA levels. A change in miRNA expression was considered statistically significant if the fold change (FC) was >2.0 and the *p*-value was <0.05.

By this, we found that miR-1-3p demonstrated the largest fold change (FC of 4.3; *p* = 0.01) at 5 min post the SS training session, based on miR-1-3p log_2_(Fold Change) values of 2,12 (Figure 3). Upregulated miR-1-3p levels were then reduced to FC = 2.2 (*p* = 0.042) at 1 h and FC = 1.8 (*p* = 0.042) at 21 h post the SS training session (Figure 3). Following the HS training session, miR-1-3p demonstrated a FC of 4.02 (*p* = 0.008) at 1 h and non-significant FC of 2.3 at 21 h, compared to baseline (Figure 3). While miR-133a-3p was significantly upregulated at 1 h post the HS training session by FC = 2.25 (*p* = 0.003) (did not reach significance at 1 h post SS training session by FC = 1.84 (*p* = 0.003)), miR-133b-3p was upregulated at 5 min and 1 h following the HS training session (FC 2.2, *p* = 0.01 and FC 2.24, *p* = 0.006, respectively) (Figure 3).

### Effect of training specific sessions on serum biomarkers

Levels of muscle damage markers serum creatine kinase (CK) and myoglobin (Mb), and the acute metabolic markers human growth hormone (hGH) and plasma lactate ([La^−^]) was also analyzed as previously described (Kristoffersen et al., 2018), and at baseline and 5 min, 1 h and 21 h post training session (Supplementary Figure S2). While serum Mb showed peak levels in cyclists 1 h after their first training session (Supplementary Figure S2), plasma [La^−^] levels and hGH levels were at its highest 5 min after a SS specific session. The range in CK levels varied largely between individuals and training session (Supplementary Figure S2).

### Correlation between miRNAs and biochemical markers following the HS and SS sessions

Post the HS session, no correlation was found between the three miRNAs and biochemical markers. 21 h post the SS session, a negative correlation was however observed between miR-133a-3p and CK (r = −0.687, *p* = 0.041) and between miR-133b-3p and [La^−^] (r = −0.711, *p* = .032).

## Discussion

The purpose of our study was to examine the acute response of a single bout of SS and HS training session in elite male cyclists, on the differential expression levels of target c-miRNAs. For this, we collected blood samples from a homogenous group of well-trained male cyclists pre- and post- SS and HS training session, investigating levels of preselected serum c-miRNAs previously associated with altered response to physical exercise by cardiac muscle function, inflammation or osteogenic differentiation, or promoting myogenesis, muscle proliferation or metabolic regulation (Dos Santos et al., 2022). In our pool of c-miRNAs, we found three out of 24 miRNAs to be significantly altered after training sessions. This c-miRNA pool most likely includes both vesicular c-miRNAs (contained in extracellular vesicles such as exosomes, microvesicles or apoptotic bodies) and nonvesicular c-miRNAs (free in circulation as part of RNA-binding protein complexes due to active export systems and/or passive leakage through the plasma membrane following cell damage or death) (Arroyo et al., 2011). Correlation between these three most differentially expressed c-miRNAs and biochemical markers [La^−^], Mb, hGH and CK was also assessed.

The acute response of a HS training session most markedly increased the abundance of miR-1-3p, miR-133a-3p and miR-133b-3p at 1 h post HS session. The SS training session most markedly increased the abundance of miR-1-3p already at 5 min post session. These three miRNAs belong to the myomiRs which are preferentially expressed in muscle, with functional roles in for instance promoting myoblast differentiation and regeneration (Horak et al., 2016), but are also detectable in human plasma (Nielsen et al., 2014). They are thought to regulate myogenesis by targeting IGF1/IGF1R and activation of the IGF1/PI3K/AKT pathway, and with a feed-back loop between IGF1 and AKT/FOXO3, influencing genes encoding myoblast precursors (Domanska-Senderowska et al., 2019).

While has-miR-1-3-p expression levels seems to mimic levels of the acute metabolic marker [La^−^] after SS training, and also mimic hGH after second bout of SS training, has-miR-133a-3p on the other hand, seems to follow the muscle damage marker Mb after first bout of HS training (Figure 2; Supplementary Figure S2). The release of Mb is highest after first bout of training during the intervention, while hGH shows highest levels after the second bout, independent of HS>SS or SS>HS. One can speculate if hGH here is affected by a metabolic stress induced during the first bout to increase after a new second bout of training. The opposite is seen for Mb, where muscles after a first bout might be in a kind of contingency for some hours after training.

Resistance training (RT) protocols, differing in the configuration of the acute program variables (e.g., muscle action, loading and volume, exercise pattern and order, rest periods, and repetition velocity and frequency), is known to cause different physiological responses (Bird et al., 2005). Due to this, there is no consensus regarding the optimal way to control such variables. A study investigating the time-course acute responses of 754 human plasma c-miRNAs to different RT protocols (strength endurance, muscular hypertrophy and maximum strength), in regular-exercise students, identified increased abundance of miR-133b 1h post maximum strength training (restored levels at 45 h) (Cui et al., 2017), and in line with our findings 1 h post HS training session. They found, however, no significant change in miR-133a levels. Another study, investigating a subset of miRNAs expressed within skeletal muscle (vastus lateralis) in healthy middle-aged men, in response to strength testing, identified also miR-133a-3p being among five of the 50 most highly expressed miRNAs (Mitchell et al., 2018).

The immediate response of a high-intensity interval exercise trial, in healthy young men, has previously identified significantly increased plasma levels of miR-1, miR-133a and miR-133b (Cui et al., 2016). These findings are somewhat in line with our observed acute response to a single bout of SS training session which significantly increased the abundance of miR-1-3p in blood serum already at 5 min post SS session. Another study investigating the response to a one-off acute sprint interval session in whole blood from healthy men found, however, this physical strain not sufficient to significantly alter miR-1-3p and miR-133a-3p levels (Denham et al., 2018). A study investigating the presence of 800 c-miRNAs in human serum versus plasma samples identified >50% of miRNAs to be uniquely present in either serum or plasma but not both, with a greater diversity of miRNAs in plasma than in serum samples (Foye et al., 2017), and indicating caution when comparing miRNA data generated from different sample types, as well as measurement platforms.

Correlation between c-miRNAs and exercise response and adaptation to a maximal incremental exercise test (VO_2_max, HR and maximum aerobic speed), and biochemical markers (CK, [La^−^]), in amateur runners, a study by Fernandez-Sanjurjo et al. (2021), identified miRNAs positively or negatively correlating with CK and [La], respectively. None of these did, however, include our miRNAs of interest (miR-1-3p, 133a-3p or 133b-3p). Our analyses showed significant negative correlations between two of our investigated miRNAs, with CK and [La], however extracting true meaningful information from these analyses, performed on such a low number of sample data that was available in this study, is difficult.

Kristoffersen et al. (2018) investigated the presence of DOMS (delayed-onset muscle soreness) post SS and HS training sessions in elite cyclists. Although they reported relatively low DOMS scores, possibly due to the higher fitness levels in the participants, a significantly higher DOMS score (and CK level) was identified 45 h after an HS session (similar levels after 21 h) compared to the SS session. In spite of this, the ability to produce power by the cyclists was found restored back to baseline already 23 h after both training sessions, indicating rapid recovery rate. Comparing this to c-miRNA levels from the same study cohort, miR-1-3p, 133a-3p and 133b-3p had all reached near baseline levels at 21 h post HS session, indicating the relevance of these myomiRs as measures of recovery post HS and SS training, opposing to the participants self-evaluation of DOMS.

There is a lack of a standard protocol for miRNA studies. The literature shows great variation in study design, source of miRNA, extraction methods, study cohort and type of training. In order to control as many variables as possible with importance to c-miRNA response and profiling in our study, the participants were of the same sex, age, had similar sleep pattern, diet, training history and physical activity before the intervention (Kristoffersen et al., 2018), and served as their own control in the intervention. Internal controls for normalization and quantification were implemented in the experimental setup. There are however several limitations to our study. Firstly, our study was based on a low number of participants in the intervention. Additional participants would most likely have provided better statistics. Secondly, we assessed miRNAs in serum samples; a sample type reported to contain lower miRNA diversity than plasma samples, possibly as a result of the coagulation process or cellular remnants (Sapp et al., 2017). Another preanalytical variable is hemolysis which can increase some miRNAs and be a confounder in these studies (McDonald et al., 2011). A hemolyzation quality control should ideally be incorporated, as well as comparing miRNA profiling in both serum and plasma. Thirdly, and due to economy, the methodology available limited the analysis to a group of pre-selected miRNAs reported to be altered after diverse acute exercises, and restrictions were imposed to the number of time-points allowed during the intervention. Future investigations might prefer global miRNA analysis, to identify novel c-miRNAs associated with training adaption and recovery or considering next-generation sequencing (NGS) as gold standard for miRNA-profiling due to greater detection sensitivity and accuracy in differential expression analysis, compared to quantitative real-time PCR. Also, longitudinal samples, collected at multiple time-points following several training sessions and longer period (months rather than hours), are likely to provide more relevant insights into the recovery and the role miRNAs might play here over time.

In conclusion our findings demonstrate that heavy strength and/or short sprint training in well-trained cyclists can increase the circulating levels of some of the miRNAs associated with muscle development. Although the roles of circulating small non-coding miRNAs are yet to be fully elucidated, our data indicate the relevance of myomiRs miR-1-3p, 133a-3p and 133b-3p as measures of acute response and recovery status.

## Data Availability

The original contributions presented in the study are included in the article/Supplementary Material, further inquiries can be directed to the corresponding author.

## Supplementary material

SUPPLEMENTARY FIGURE S1 Unsupervised hierarchical clustering analysis of miRNAs in serum. The heat map illustrates the result of the one-way hierarchical clustering of 24 miRNAs, including endogenous- and spike-in controls. Samples derived from 9 cyclists were analyzed at all timepoints pre-/post SS and HS training sessions, using the TLDA assay. Each row represents one miRNA and each column represents one sample. Similarities between samples were analyzed by Euclidean Distance, complete linkage method, and map type global (ΔCT), using the ExpressionSuite software. * denotes spike-in controls; ** denotes endogenous controls; CTL indicates mandatory array card control (cel-miR-39-3p). The color scale shown to the left illustrates the relative expression levels of miRNA across all samples: red color represents expression level above the mean, blue color represents expression level lower than the mean (confidence level 95%). Cyclists and training session time points (IDs) are shown horizontally below each figure. Clustering was performed on **(A)** all miRNAs and **(B)** miRNAs that have significant different expression level with *p* < 0.05.

SUPPLEMENTARY FIGURE S2 Dot-plots of biochemical marker levels in serum following cyclists’ training sessions. Changes in levels of creatine kinase (CK) **(A, B)**, plasma lactate (La^-^) **(C, D)**, serum myoglobin (Mb) **(E, F)**, and human growth hormone (hGH) **(G, H)**, following a heavy strength training session and thereafter a short sprint session (HS>SS) **(A, C, E, G)**, or following a short sprint training session and thereafter a heavy strength session (SS>HS) **(B, D, F, H)**. Biochemical marker levels were measured at baseline and post-exercise (5 min, 1 h, 21 h and 45 h/baseline) in nine well-trained cyclists. Mean is shown as a horizontal line.
