# Supplementary material for: Acute response in circulating microRNAs following a single bout of short-sprint and heavy strength training in well-trained cyclists
Source: Front Physiol. 2024 Mar 12;15:1365357. doi: 10.3389/fphys.2024.1365357 (PMC10963392; doi:10.3389/fphys.2024.1365357)
Supplement: Supplementary file 1 [file Table1.DOCX]

**Supplementary Table 1.** 24 different human miRNAs screened and listed according to expected functional regulation.

| **Functional regulation** | **miRNA** | **Assay name** | **Assay ID** | **Reference** |
| --- | --- | --- | --- | --- |
| Skeletal and hearth muscle | 1-3p | hsa-miR-1-3p | 477820_mir | Siracusa J, et al., 2018;  Domanska-Senderowska D et al., 2019 |
|  | 20a-5p | hsa-miR-20a-5p | 478568_mir | Baggish AL, et al., 2011;  Aoi, W, et al., 2013 |
|  | 21-5p | hsa-miR-21-5p* | 477975_mir | Baggish AL, et al., 2011;  Wardle SL, et al., 2015 |
|  | 29b-3p | hsa-miR-29b-3p | 478369_mir | Nielsen S, et al. 2014 |
|  | 124-3p | hsa-miR-124-3p | 477879_mir | McCarty JJ, 2011 |
|  | 133a-3p | hsa-miR-133a-3p | 478511_mir | Nielsen S, et al. 2014;  Siracusa J, et al., 2018 |
|  | 133b-3p | hsa-miR-133b-3p | 480871_mir | Sawada S, et al., 2013;  Domanska-Senderowska D et al., 2019 |
|  | 206-3p | hsa-miR-206-3p | 477968_mir | Xu T, et al., 2015;  Siracusa J et al., 2018 |
|  | 208a-3p | hsa-miR-208a-3p | 477819_mir | Siracusa J, et al., 2018 |
|  | 208b-3p | hsa-miR-208b-3p | 477806_mir | Siracusa J, et al., 2018 |
|  | 486-3p | hsa-miR-486-3p | 478422_mir | Aoi, W, et al., 2013 |
|  | 486-5p | hsa-miR-486-5p | 476128_mir | Siracusa J, et al., 2018 |
| Hypoxia and angiogenesis | 20a-5p | hsa-miR-20a-5p | 478568_mir | Baggish AL, et al., 2011 |
|  | 126-3p | hsa-miR-126-3p | 477887_mir | Baggish AL, et al., 2011 |
|  | 146a-5p | hsa-miR-146a-5p | 478399_mir | Aoi, W, et al., 2013 |
|  | 210-3p | hsa-miR-210-3p | 477970_mir | Gao, J et al. 2023 |
|  | 221-3p | hsa-miR-221-3p | 477981_mir | Wardle SL, et al., 2015;  Liu X, et al., 2009 |
|  | 222-3p | hsa-miR-222-3p | 477982_mir | Baggish AL, et al., 2011;  Liu X, et al., 2009 |
| Metabolic | 23a-3p | hsa-miR-23a-3p | 478532_mir | Ceccarelli G, et al, 2017 |
|  | 122-5p | hsa-miR-122-5p | 477879_mir | Lou J, et al., 2022 |
|  | 140-5p | hsa-miR-140-5p | 477909_mir | Mayr B et al., 2018 |
|  | 181a-5p | hsa-miR-181-5p | 477857_mir | Aoi W, et al., 2014;  Russel AP, et al. 2013 |
| Inflammation | 21-5p | hsa-miR-21-5p* | 477975_mir | Baggish AL, et al., 2011 |
| Somatic | 16-5p | hsa-miR-16-5p | 477860_mir | Cui SF, et al, 2015 |
|  | 122-3p | hsa-miR-122-3p | 477874_mir | Cui SF, et al, 2015 |
|  | 149-5p | hsa-miR-149-5p | 477917_mir | Sawada S et al, 2013 |

*****hsa**-**miR 21-5p is listed as both a myomiR and involved in inflammation.

*hsa**-**miR 20a-5p is listed as both a myomiR and involved in hypoxia and angiogenesis.
