# Supplementary figures and images for: Acute response in circulating microRNAs following a single bout of short-sprint and heavy strength training in well-trained cyclists
Source: Front Physiol. 2024 Mar 12;15:1365357. doi: 10.3389/fphys.2024.1365357 (PMC10963392; doi:10.3389/fphys.2024.1365357)

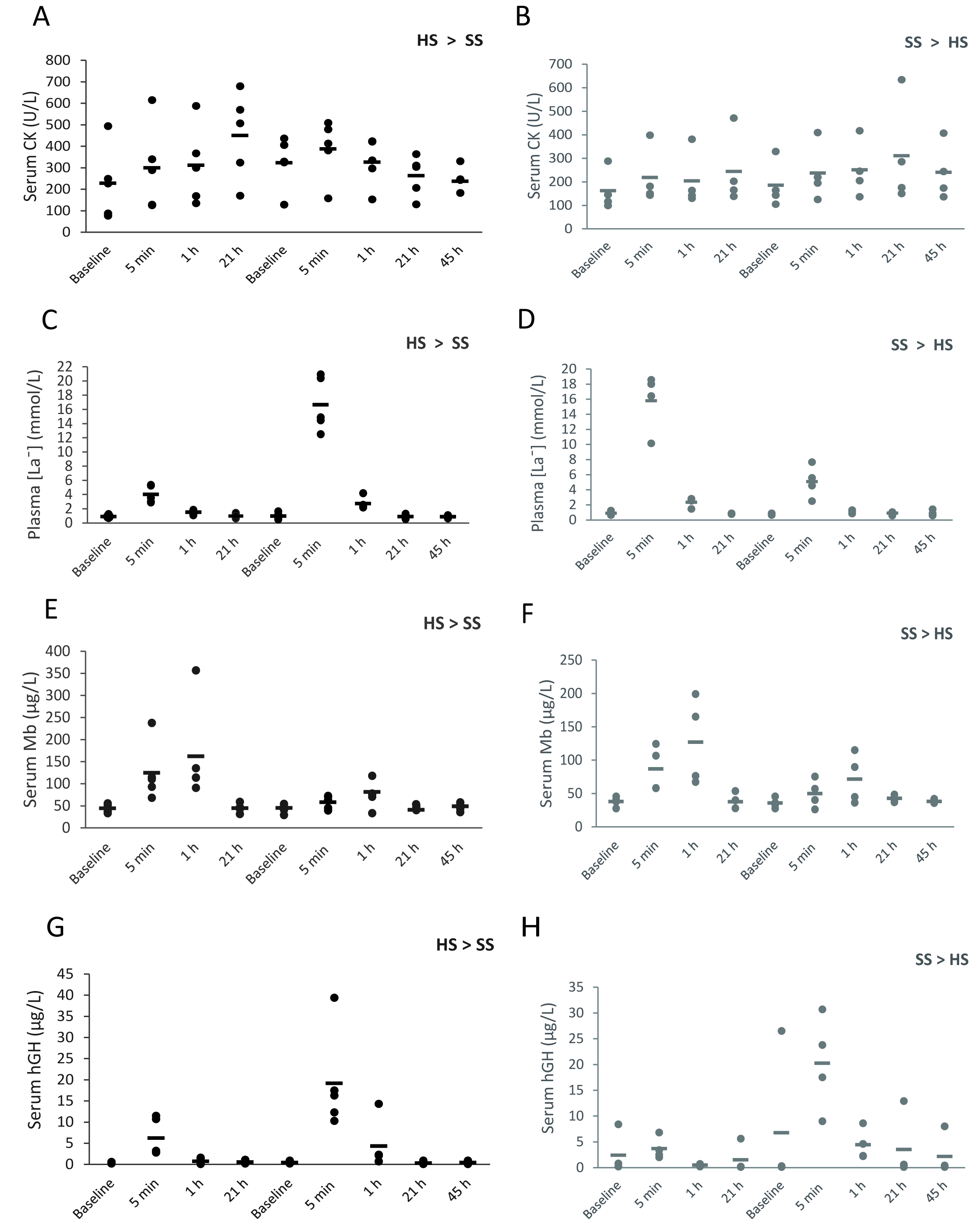

Supplement: Supplementary file 2 [file Image2.TIF]

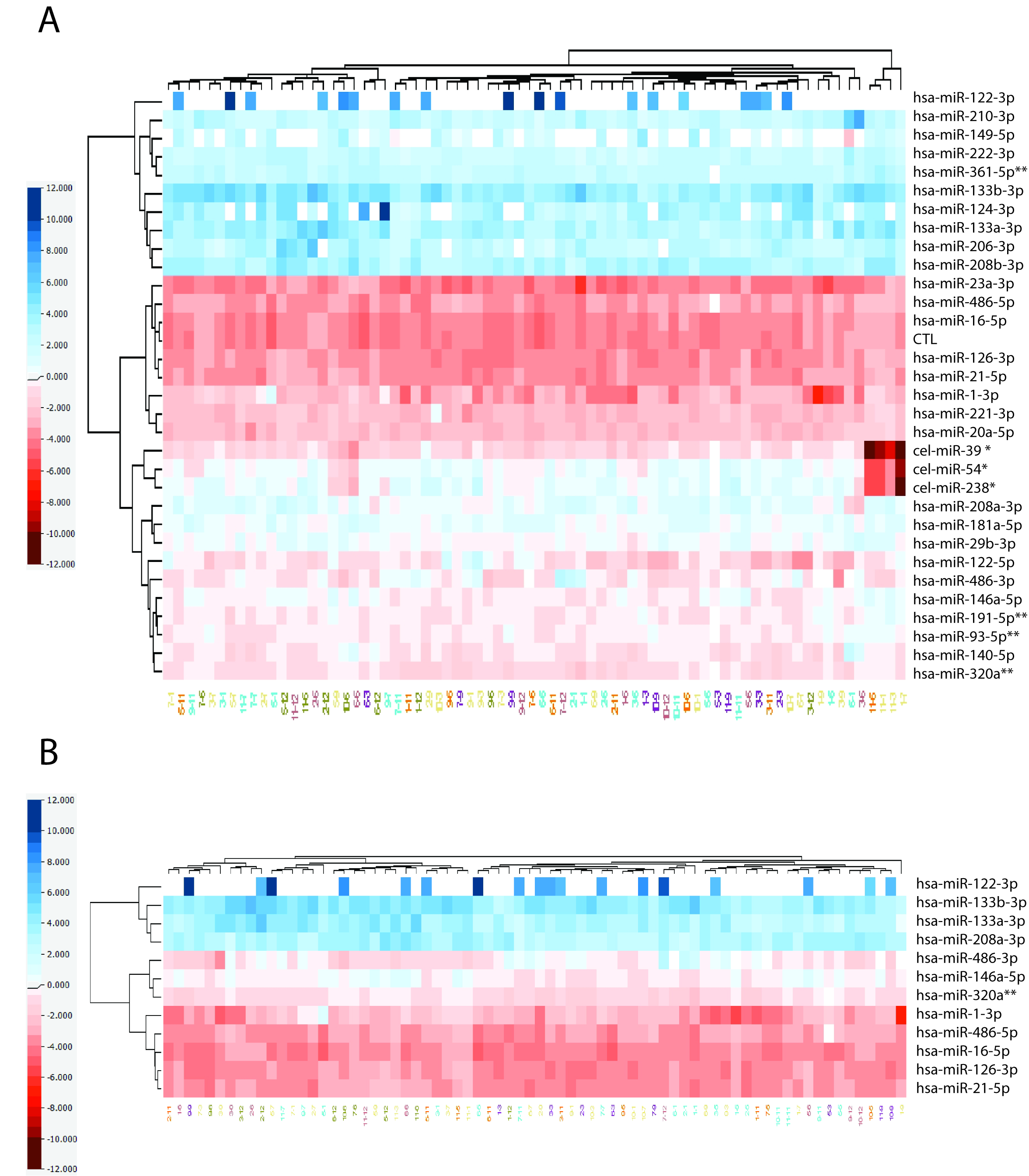

Supplement: Supplementary file 3 [file Image1.TIF]
